# Regular Consumption of Green Tea as an Element of Diet Therapy in Drug-Induced Liver Injury (DILI)

**DOI:** 10.3390/nu16172837

**Published:** 2024-08-24

**Authors:** Anna Winiarska-Mieczan, Karolina Jachimowicz-Rogowska, Małgorzata Kwiecień, Marta Borsuk-Stanulewicz, Agnieszka Tomczyk-Warunek, Ewa Stamirowska-Krzaczek, Cezary Purwin, Małgorzata Stryjecka, Marzena Tomaszewska

**Affiliations:** 1 Institute of Animal Nutrition and Bromatology, Department of Bromatology and Nutrition Physiology, University of Life Sciences in Lublin, Akademicka 13, 20-950 Lublin, Poland; karolina.jachimowicz@up.lublin.pl (K.J.-R.); malgorzata.kwiecien@up.lublin.pl (M.K.); 2 Institute of Human Nutrition and Agriculture, The University College of Applied Sciences in Chełm, Pocztowa 54, 22-100 Chełm, Poland; ekrzaczek@panschelm.edu.pl (E.S.-K.); mstryjecka@panschelm.edu.pl (M.S.); mtomaszewska@panschelm.edu.pl (M.T.); 3 Department of Animal Nutrition and Feed Science, University of Warmia and Mazury in Olsztyn, 10-719 Olsztyn, Poland; marta.borsuk@uwm.edu.pl (M.B.-S.); purwin@uwm.edu.pl (C.P.); 4 Laboratory of Locomotor Systems Research, Department of Rehabilitation and Physiotherapy, Medical University of Lublin, Jaczewskiego 8, 20-954 Lublin, Poland; a.tomczykwarunek@gmail.com

**Keywords:** DILI, liver, green tea, catechin, metabolism of drugs, microbiota

## Abstract

The liver is a highly metabolically active organ, and one of the causes of its dysfunction is the damage caused by drugs and their metabolites as well as dietary supplements and herbal preparations. A common feature of such damage is drugs, which allows it to be defined as drug-induced liver injury (DILI). In this review, we analysed available research findings in the global literature regarding the effects of green tea and/or its phenolic compounds on liver function in the context of protective action during prolonged exposure to xenobiotics. We focused on the direct detoxifying action of epigallocatechin gallate (EGCG) in the liver, the impact of EGCG on gut microbiota, and the influence of microbiota on liver health. We used 127 scientific research publications published between 2014 and 2024. Improving the effectiveness of DILI detection is essential to enhance the safety of patients at risk of liver damage and to develop methods for assessing the potential hepatotoxicity of a drug during the research phase. Often, drugs cannot be eliminated, but appropriate nutrition can strengthen the body and liver, which may mitigate adverse changes resulting from DILI. Polyphenols are promising owing to their strong antioxidant and anti-inflammatory properties as well as their prebiotic effects. Notably, EGCG is found in green tea. The results of the studies presented by various authors are very promising, although not without uncertainties. Therefore, future research should focus on elucidating the therapeutic and preventive mechanisms of polyphenols in the context of liver health through the functioning of gut microbiota affecting overall health, with particular emphasis on epigenetic pathways.

## 1. Introduction

The liver is an important gland of the digestive system and one of the most essential organs for the entire body, as it performs many critical functions necessary for its proper functioning. The liver has several functions, including filtration, storage, detoxification, and metabolism, which complement each other [1]. The detoxification function of the biotransformation of xenobiotics is crucial for maintaining homeostasis in the body. Any substance that is not utilised by the body is excreted quickly. This was facilitated by the biphasic metabolism of xenobiotics. Because the liver performs many critical functions in the body, its damage is the cause of many diseases, sometimes with serious consequences [1,2,3].

Given that the liver is a highly metabolically active organ, one of the causes of liver dysfunction is damage due to drugs and their metabolites as well as dietary supplements and herbal preparations [4]. A common feature of such damage is its cause, which is, the drug, which allows it to be classified as drug-induced liver injury (DILI). DILI accounts for approximately 10% of all cases of acute hepatitis, causes acute jaundice in 50% of patients with new jaundice, and is responsible for approximately 50% of cases of acute liver failure [5]. The pathogenesis of DILI is highly complex and is not yet fully understood. Various drugs and/or their toxic metabolites can directly affect liver cells, as well as induce inflammation, oxidative stress, and mitochondrial damage [6,7,8,9,10]. Often, drugs cannot be eliminated, but appropriate nutrition can strengthen the body and the liver, which may mitigate adverse changes resulting from DILI. Some dietary components, such as phenolic compounds, exhibit antioxidant and anti-inflammatory effects [11]. These are found in many unprocessed or minimally processed plant-based foods, such as tea [11,12]. Tea can be consumed widely, regardless of age and physiological conditions, making it an excellent candidate as a fundamental element of diet therapy in DILI. In particular, green tea deserves special attention because it contains the highest amount of catechins, primarily epigallocatechin gallate (EGCG), which exhibits strong hepatoprotective effects [13,14].

In this review, we analyse the available global literature on the effects of green tea and/or its phenolic compounds on liver function in the context of protective action during prolonged exposure to xenobiotics. We focused on both the direct detoxifying effect of EGCG in the liver and the impact of EGCG on the gut microbiota and its influence on liver health. The role of the gut microbiota in the metabolism of EGCG and its impact on liver function is a unique aspect of this text. This highlights the complex relationships between gut flora, food components, and their influence on physiological functions. Strengthening liver health can directly translate into mitigating DILI symptoms and reducing the risk of disease in individuals taking high doses of pharmaceutical agents.

## 2. Methodology of Information Retrieval in Databases

**Figure 1:**
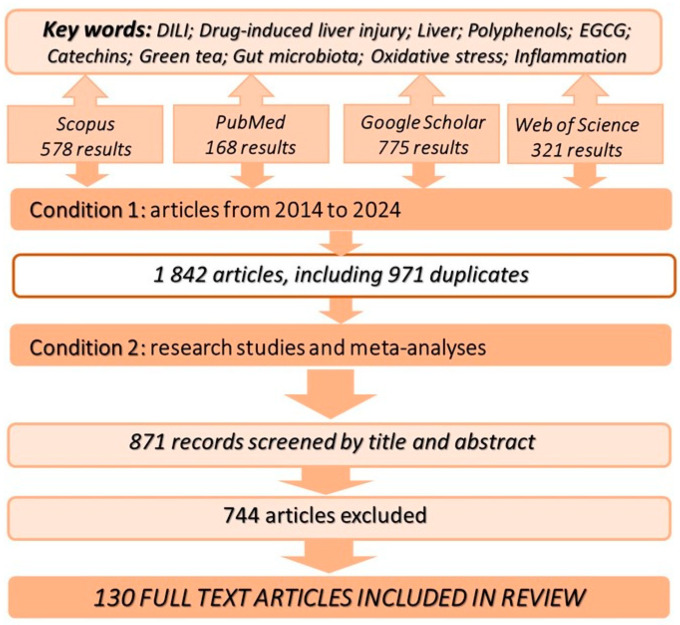
Methodology for reviewing the existing literature.

During the preparation of this review, scientific information available in the Scopus, PubMed, Web of Science, and Google Scholar databases was analysed. The analysis was conducted in May 2024. The following keywords were used: “DILI”, “drug-induced liver injury”, “liver”, “polyphenols”, “EGCG”, “catechins”, “green tea”, “gut microbiota”, “oxidative stress”, and “inflammation”, in both Polish and English (Figure 1). Titles and abstracts were screened, and articles not meeting the substantive criteria (Condition 1: articles from 2014 to 2024, and Condition 2: research studies and meta-analyses), specifically those that did not include results from studies conducted on laboratory animals or involving humans, were excluded. Only research studies and meta-analyses have been conducted to date. In the case of meta-analyses, duplicate data were used only once (source texts were referenced).

Articles that met these criteria underwent a more detailed analysis to identify the most significant publications and to exclude duplicates. Additionally, the bibliographies of all selected articles were reviewed to uncover any further potentially relevant texts. The search was restricted to studies published between 2014 and 2024. In total, 1842 publications were analyzed, with 130 being included in the final manuscript. The protocol was developed following the guidelines outlined in the Preferred Reporting Items for Systematic Reviews and Meta-Analysis Protocols (PRISMA-P) 2015 statement.

## 3. Pathophysiological Mechanisms of DILI

**Figure 2:**
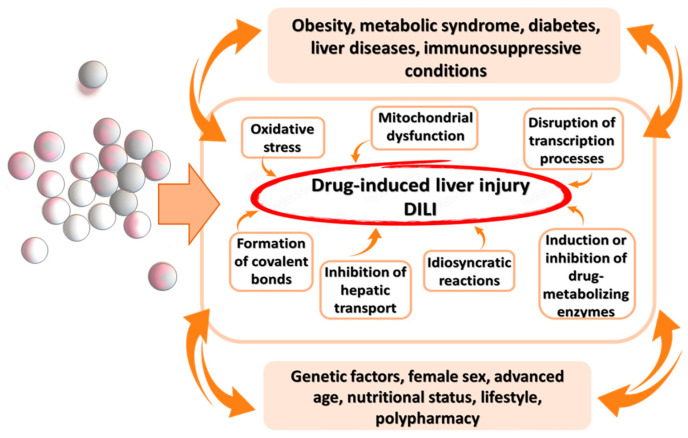
Pathophysiological mechanisms of DILI.

DILI is a complex condition characterised by liver damage caused by the administration of drugs and their metabolites. The pathophysiological mechanisms underlying DILI are multifaceted (Figure 2). However, several key processes have been identified: direct toxic effects of drugs, oxidative stress, inflammation, mitochondrial damage, and immune reactions [2,6,8,9,10]. The most common cause of DILI is the use of antimicrobial agents, herbal and dietary supplements, and medicinal preparations [15,16,17]. According to some data, over 50% of acetaminophen overdose cases result in acute liver failure and approximately 20% lead to liver transplantation [18]. The use of high doses of acetaminophen leads to the accumulation of the reactive metabolite *N*-acetyl-p-benzoquinone imine (NAPQI), which causes liver cell damage leading to necrosis and resulting in liver failure [18].

Among the biochemical disturbances accompanying DILI, the most characteristic, although non-specific, indicator of liver parenchymal damage is an increase in alanine aminotransferase (ALT) activity > 200 U/L, aspartate aminotransferase (AST) >200 U/L, total bilirubin > 3.0 mg/dL, or international normalised ratio (INR > 3). Other indicators include oliguria, creatinine > 200 micromol/L, acidosis (pH < 7.3), and lactate > levels > 3 mmol/L [19,20]. Genetic factors, female sex, advanced age, nutritional status, lifestyle (smoking, alcohol, and drugs), and polypharmacy (exposure to multiple drugs simultaneously) predispose individuals to DILI [20,21]. Concomitant metabolic diseases such as obesity, metabolic syndrome, diabetes, liver diseases, and immunosuppressive conditions also play significant roles [19,20].

There are three types of DILI: hepatocellular, cholestatic, and mixed [7,22]. The pathogenesis of DILI is highly complex and not yet fully understood. However, recent studies using improved preclinical models have provided better insights into the mechanisms underlying drug-induced liver injury. Various drugs and their toxic metabolites can directly affect liver cells and cause inflammation, oxidative stress, and mitochondrial damage. Drugs can induce liver cell damage through different molecular pathways, including direct hepatotoxicity and immune responses. Consequently, various pathomechanisms of DILI can be distinguished [6,7,8,9,10]: (1) Induction of oxidative stress: this causes the generation of large amounts of reactive oxygen species (ROS), which activate Kupffer cells; (2) mitochondrial dysfunction: resulting from oxidative stress, leading to impaired energy production and increased cell damage; (3) disruption of transcription processes: due to oxidative stress, affecting normal cell functions and survival; (4) formation of covalent bonds: leading to loss of function and/or autoimmune reactions; inhibition of hepatic transport: affecting the normal excretion and processing of substances in the liver; (5) induction or inhibition of drug-metabolising enzymes: affecting how drugs are processed and potentially leading to toxic accumulations; and (6) idiosyncratic reactions: unpredictable reactions that are not dose-dependent and occur only in susceptible individuals. These mechanisms highlight the diverse ways in which drugs can induce liver damage, underscoring the need for a nuanced understanding of the pathogenesis of DILI to develop effective preventive and therapeutic strategies. The metabolism (biotransformation) of drugs in the body involves two phases: (1) Phase I reactions, which modify the chemical structure of the drug through oxidation, reduction, and hydrolysis; and (2) Phase II reactions, which involve conjugating the Phase I metabolite with active acetate CoA, 3′-phosphoadenosine-5′-phosphosulfate, glucuronic acid, and amino acids [23,24].

Lipid peroxidation is a major pathogenic factor that triggers hepatocyte necrosis and inflammatory processes in liver diseases [25]. Mitochondrial oxidative stress alone is insufficient to induce hepatocyte cell death; however, this process is stimulated by mitogen-activated protein kinases (MAPKs), leading to increased expression of the JNK genes JNK1 and JNK2 in the liver [6,26,27]. The combined activities of JNK1 and JNK2 in hepatocytes protect against toxic liver injury, as demonstrated in studies using mice intoxicated with CCl4 and acetaminophen as well as in vitro studies using human hepatocytes [6].

## 4. Effect of Gut Microbiota on Liver Function

**Figure 3:**
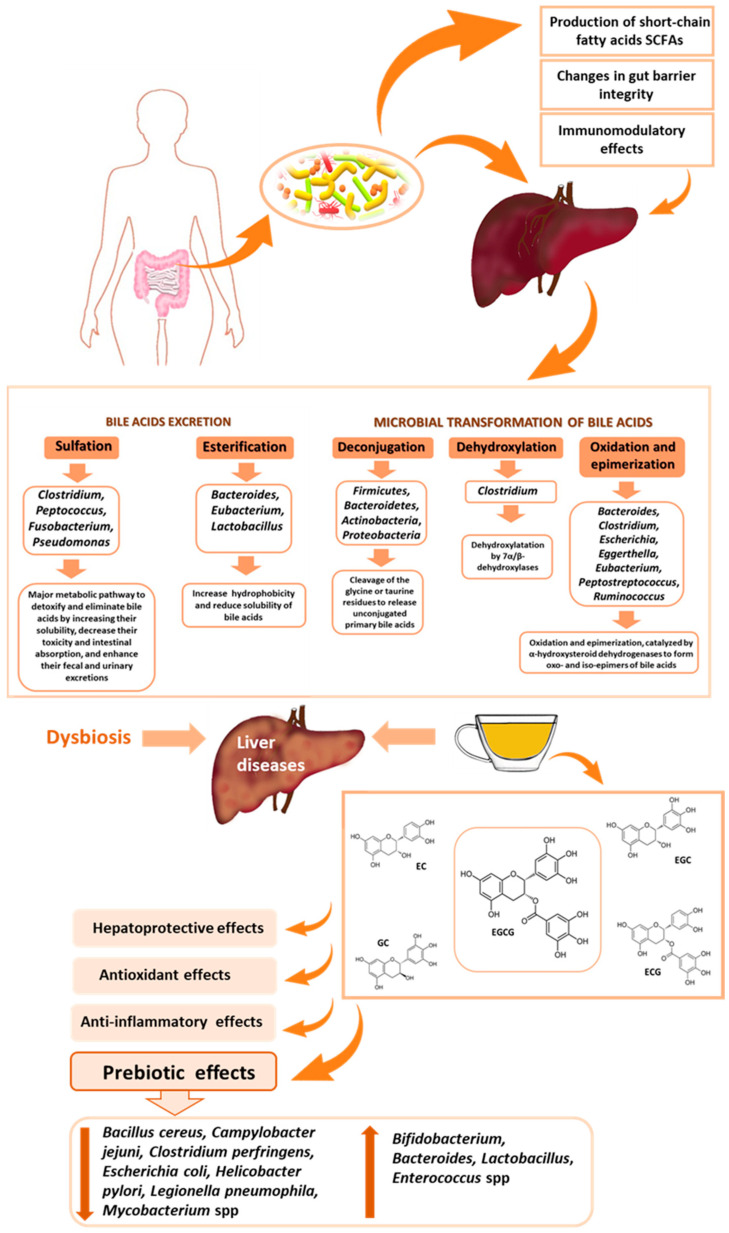
Relationship between gut microbiota, tea, and liver health.

The gut and liver are closely interconnected and communicate via the portal vein and bile duct system, exposing the liver to bacterial products and metabolites originating from the intestine [28]. Gut microbiota can influence liver function and health through various mechanisms: (1) Production of metabolites: gut microbiota ferment dietary, producing short-chain fatty acids (SCFAs) such as acetic acid, propionic acid, and butyric acid [29]; SCFAs help maintain gut integrity by stimulating mucus production and by forming a protective barrier between the gut lumen and epithelial cells [30]; (2) changes in gut barrier integrity: dysbiosis can lead to dysfunction of the gut barrier, allowing microbial components to translocate to the liver and contributing to liver diseases [29,30,31,32,33,34]; (3) immunomodulatory effects: microbiota-derived metabolites regulate the function and efficiency of the immune system, and diet plays a key role in shaping the composition and function of the gut–liver axis microbiota [35]; chronic inflammation triggered by microbiota dysbiosis and resulting immune dysregulation affects the development of chronic liver diseases [36,37,38,39]; and (4) impact on bile acid metabolism: bile acids are synthesised in the liver from cholesterol and undergo biotransformation by the gut microbiota in the small intestine (Figure 3), involving major gut microbiota groups such as *Firmicutes*, *Bacteroidetes*, *Actinobacteria*, and *Proteobacteria* [40,41,42,43].

Gut microbiota can influence liver function and health through various mechanisms: production of metabolites, changes in gut barrier integrity, immunomodulatory effects, and impact on bile acid metabolism. Bile acids are synthesised in the liver from cholesterol and undergo biotransformation by the gut microbiota in the small intestine, involving major gut microbiota groups such as *Firmicutes, Bacteroidetes, Actinobacteria*, and *Proteobacteria*. Primary bile acids conjugated in the liver are deconjugated by the microbiota, leading to the formation of glycine, taurine, and deconjugated primary bile acids, which are further metabolised into secondary bile acids and excreted from the body. The gut microbiota regulates bile acid metabolism by lowering the levels of tauro-β-muricholic acid, a natural antagonist of the farnesoid X receptor (FXR), and inhibiting bile acid synthesis in the liver by alleviating FXR inhibition in the ileum [40,41]. In turn, bile acids and their metabolites can modulate gut microbiota composition by exerting antimicrobial effects and activating host signalling pathways [42]. Studies have shown that Gram-negative bacteria are more resistant to bile acids than Gram-positive bacteria, although the results have not been entirely consistent [43]. It has also been demonstrated that unconjugated bile acids have stronger antibacterial activities than conjugated bile acids [42,43].

Structural changes in the microbiota led to modifications in metabolite production, possibly contributing to liver disease progression [36,37,38,39]. Studies have indicated that dietary factors influence microbiota composition, metabolite production, and gut permeability [44,45,46]. Therefore, modulating the gut microbiota composition and/or its metabolites through bioactive food components could be considered a potential dietary therapy approach for liver diseases, including DILI.

## 5. The Potential of Green Tea as a Component of Dietotherapy in DILI

Risk factors for DILI, besides the use of large quantities of pharmaceutical agents, also include several factors influencing the body’s condition. Most commonly mentioned are oxidative stress, inflammation, and chronic diseases, with oxidative stress and inflammation being markers for conditions such as obesity, metabolic syndrome, and diabetes [7,47]. Some active substances present in food have properties that can reduce the risk of these factors. Green tea exhibits antioxidant, anti-inflammatory, anti-obesogenic, and antidiabetic properties [11,12,48,49]. Additionally, its hepatoprotective effects have been demonstrated (Table 1 and Table 2). The presence of phenolic compounds, notably catechins such as EGCG, contributes to this, as they are major secondary metabolites in tea and frequently serve as indicators of its quality [12,48,50]. Therefore, green tea can be considered as a component of dietotherapy for liver diseases, including DILI. However, it is important to note the controversies associated with consuming excessive amounts of green tea, primarily because of the presence of EGCG.

### 5.1. Phenolic Compounds in Green Tea

In tea leaves, three primary groups of polyphenols can be distinguished: catechins (EGCG, epicatechin EC, epigallocatechin EGC, epicatechin gallate ECG, gallocatechin GC, and gallate gallate GCG), theaflavins, and thearubigins. The content of polyphenols is influenced by various factors such as tea type, origin, tea-growing region, and the age of the plant; as the plant ages, the content of phenolic compounds in the leaves decreases [12,70]. Green tea infusion contains approximately 0.2–0.5 mg total phenolic content (TPC), 5–8.5 µg flavonoids, and around 0.08–0.1 µg anthocyanins [12]. Tea leaves also contain tannins, which are products of polyphenol oxidation [11]. Phenolic compounds are characterised by low bioavailability in the small intestine [71], and their absorption involves the gut microbiota.

The phenolic content and antioxidant potential of tea infusions are significantly influenced by the brewing method [12,72]. Studies have shown that phenolic compounds are relatively thermostable; at temperatures of 60, 80, and 100 °C, degradation ranges from 15% to 30% after 4 h of exposure [73]. More than half of the polyphenols are extracted into the infusion during the initial 5 min of brewing [72,74]. Research has demonstrated that extending the brewing time of loose-leaf green tea from 5 to 10 min increases the TPC in the infusions by 42% and further by an additional 12% up to 15 min [12]. For green tea in bags, this increase was 35% and 37%, respectively [12]. It is believed that the polyphenols in the tea infusion are responsible for its health benefits. Therefore, longer brewing times are more advantageous for consumers. However, an excessive content of polyphenols, especially tannins, can cause overly bitter infusions, thereby reducing consumer appeal [75,76].

### 5.2. Hepatoprotective Effects of Green Tea

The hepatoprotective effect of green tea involves preventing cell apoptosis; regulating ALT, AST, and ALP activities; and tumour necrosis factor-alpha (TNF-α), as well as enhancing antioxidant potential (Table 1 and Table 2). Such effects were observed in rats with liver fibrosis induced by thioacetamide (inhibiting liver fibrosis progression, reducing proliferating cell nuclear antigen PCNA, preventing hepatocyte oxidation, increasing superoxide dismutase SOD activity, catalase CAT, decreasing malondialdehyde MDA, and reducing liver cell inflammation) [63] and in rats intoxicated with cyclophosphamide (mildly enlarged portal vein, mild periportal mononuclear cell infiltration, periportal and portal collagen fibre formation) [77]. In mice with acute liver damage induced by carbon tetrachloride CCl4, green tea (200, 400, and 800 mg/kg, twice per day for 7 days) exhibited hepatoprotective effects by improving the liver antioxidant status and preventing cell apoptosis through caspase-3-dependent signalling pathways [78]. The use of green tea extract in mice subsequently poisoned with CCl4 confirmed the hepatoprotective action of tea, as evidenced by decreased serum ALT, AST, and ALP activities; inhibition of liver MDA levels; improved hepatosomatic index profile; protection against histological changes; and increased glutathione peroxidase (GPX) and SOD activities compared to mice not receiving tea before CCl4 exposure [50]. A study conducted by Wang et al. [49] aimed to elucidate the potential anti-fibrogenic role of three abundant tea catechins (ECG, EGC, and EGCG) in CCl_4_-induced liver fibrosis in rats and the underlying molecular mechanisms. Catechins effectively improved oxidative state parameters and liver histology as well as alleviated liver fibrosis (reduced desmin expression, α-smooth muscle actin, transforming growth factor-beta (TGF-β), and ERK1/2 and Smad1/2 phosphorylation). The administration of green tea polyphenols (400 mg/kg body weight) to rats poisoned with cadmium sulphate (50 mg CdSO_4_ per L) for 30 days led to a sharp decrease in serum ALT and AST activities as well as improved liver architecture, preventing Cd-induced steatosis and hepatocyte necrosis [79]. Oral administration of green tea extract to Pb-poisoned rats for 4 weeks resulted in improved serum ALT, AST, and ALP activity [80]. Furthermore, consuming green tea extract during lactation reduces hepatic lipid accumulation in rats exposed to a high-fat diet from prenatal to adulthood [81]. Providing tea flower extract to mice showed hepatoprotective effects, including protection against *Propionibacterium acnes* and lipopolysaccharide-induced liver inflammation, reversing histological damage and serum ALT elevation and lowering levels of nitric oxide (NO), TNF-α, and IL-1β mRNA in mice with immune-mediated liver inflammation [82]. The hepatoprotective potential of green tea in preventing diet-induced fatty liver was observed in studies conducted on Wistar rats [83] and mice, which exhibited reduced liver steatosis, decreased hypertriglyceridemia and hyperglycaemia, and improved insulin resistance [84]. Positive outcomes were achieved through the activation of the sirtuin 1 protein kinase and AMP pathways [84]. The cited studies highlighted that EGCG is responsible for the positive results obtained.

EGCG alleviates hepatocyte damage and dysfunction by mitigating resistance to FGF21 (fibroblast growth factor-21) and enhancing the FGFR/AMPK pathway, thereby alleviating oxidative stress as observed in mice fed a high-fat diet [85]. EGCG effectively mitigated liver damage and mitochondrial dysfunction caused by acetaminophen in laboratory animals [13,14]. The hepatoprotective ability of EGCG stems from its antioxidant properties, which improve membrane potential and respiratory chain complex activity in liver mitochondria, thus preventing mitochondrial dysfunction [13,86]. Studies on rats exposed to CCl4 have shown that EGCG exerts protective effects on the liver through its antioxidative actions (reducing MDA levels and increasing GSH), anti-inflammatory effects (lowering levels of inflammatory markers: TNF-α, NF-κB, IL-1β, and TGFβ), and antifibrotic effects (reducing fibrotic markers: *p*-ERK and *p*-Smad1/2 protein expression) [87]. It has been observed that (−)-epigallocatechin 3-O-(3-O-methyl) gallate (EGCG3″Me) effectively mitigates alcohol-induced liver changes in a concentration-dependent manner in mice [88]. Administration of EGCG3″Me at a dose of 100 mg/kg BW per day significantly reduced serum AST and ALT levels and liver MDA levels and restored SOD and GPX activities. In an in vitro study using human liver cells (HL-7702), EGCG reversed Cd-induced reduction in cell viability and apoptosis, likely due to ROS scavenging and maintenance of redox homeostasis [89]. Studies in mice have identified EGCG as a potential inhibitor of ferroptosis by increasing FTH/L (Ferritin H/L), NRF2 (nuclear factor erythroid 2-related factor 2), and GPX4 expression, suggesting that it could be considered a therapeutic agent for liver diseases induced by iron overload [90].

### 5.3. The Antioxidant Action of Green Tea

The antioxidant effects of tea are largely due to its polyphenolic components, such as catechins, quercetin, theaflavins, thearubigins, and tannic acid, which act through [11,91] (1) the inhibition of oxidative enzyme activity and chelation of trace elements; (2) scavenging of ROS; (3) enhancement of endogenous enzymatic and non-enzymatic antioxidant activities; and (4) ability to neutralise singlet oxygen by donating electrons or hydrogen atoms.

Green and white teas are characterised by the highest antioxidant capacity (total phenolic, flavonoid and caffeine content, ferric-reducing antioxidant power, DPPH radical scavenging capacity) [11,92]; although, the antioxidant activity of tea infusions depends on brewing time, which is 15 min for green tea [12]. It has been shown that green tea infusions strongly inhibit linoleic acid peroxidation in mice [93]. Increased antioxidant potential due to green tea infusion has been observed in the blood serum of rats exposed to Cd (7 mg/kg feed) and Pb (50 mg/kg feed) [11]. Green tea (1.5% aqueous solution) positively affected rats with tamoxifen-induced liver damage (45 mg/kg/day), as evidenced by increased CAT, SOD, and GPX activity [94]. Oral administration of green tea extract to Pb-poisoned rats for 4 weeks significantly improved the antioxidant parameters glutathione S-transferase (GST) and SOD in the liver [80]. Lv et al. [95], through in silico and in vivo studies, demonstrated that green tea can activate the Nrf2 signalling pathway in mouse livers by disrupting the Nrf2-Keap1 protein–protein interaction, explaining green tea’s antioxidant action, with EGCG showing the highest activity. The transcription factor Nrf2 controls the expression of genes containing antioxidant response element sequences in their promoters and encodes enzymes such as GST, NAD(P)H quinone dehydrogenase 1, heme oxygenase 1 (HO-1), and γ-glutamylcysteine synthetase (γ-GCS) [96].

### 5.4. Anti-Inflammatory Action of Green Tea

In vitro studies have demonstrated significant anti-inflammatory effects of aqueous extracts of green tea leaves, which have been attributed to their high flavonoid content [97]. Flavonol glycosides and aglycones from green tea markedly reduced mRNA expression of inflammation-related genes dose-dependently in an in vitro study using mouse macrophages RAW 264.7 [98]. Obese diabetic mice treated with green tea extract showed reduced release of soluble intercellular adhesion molecule-1 (sICAM-1), indicating the tea’s anti-inflammatory properties [99]. The anti-inflammatory effect of green tea was also observed in a study involving obese women, where increased adiponectin activity and decreased high-sensitivity C-reactive protein (hs-CRP) levels were noted [100]. Similarly, in obese men, green tea consumption led to a decrease in IL-6 and hs-CRP levels and an increase in adiponectin [101]. The anti-inflammatory properties of green tea have been further confirmed in studies conducted on mice [102] and rats [103].

The impact of tea polyphenols on inflammatory processes involves inhibition of pro-inflammatory cytokine synthesis, IFN-γ, TNF-α, NF-κB, and chemokines in cells, as well as regulation of mitogen-activated protein kinase (MAPK), inducible nitric oxide synthase (iNOS), HO-1, arachidonic acid, cyclooxygenase-2 (COX-2), and lipoxygenase (LOX) [104,105,106,107,108]. EGCG inhibits NF-κB and MAPK activation, reduces IFNγ, TNF-α, and IL-1β expression, stimulates genes related to immunity (e.g., TNF-α, MAPK, NOS), and inhibits apoptosis [109]. EGCG can also inhibit inflammatory leukocyte infiltration and pro-inflammatory IL-8, and studies in mice have shown it decreases NF-κB and IL-6 inflammatory actions [109,110,111].

### 5.5. Anti-Obesogenic and Anti-Diabetic Effects of Green Tea

Green tea contains numerous biologically active substances that facilitate weight loss by [112,113,114] (1) suppressing appetite by stimulating noradrenaline production and activating the sympathetic nervous system, which induces a feeling of fullness; (2) reduction in the metabolic rate, resulting in an increase in energy expenditure; (3) enhancing lipid metabolism through adrenergic receptor activation; and (4) inhibiting pancreatic lipase production by covalently binding to serine in the enzyme’s active site.

Active substances in tea that facilitate weight loss include polyphenols, flavonoids, caffeine, caffeic acid, and chlorogenic acid [115].

Its anti-diabetic properties are primarily exhibited by polyphenols, catechins (especially EGCG), gallic acid, caffeine, theaflavin, and polysaccharides. These compounds regulate blood glucose levels by [116,117] (1) reducing ROS levels; (2) inhibiting α-amylase and α-glucosidase activity, with inhibitory activity dependent on the amount of hydroxyl groups in the compound; and (3) modulating the expression of pro-inflammatory cytokines that may decrease glucose-induced insulin secretion.

Studies in obese diabetic mice have shown that green tea extract has anti-diabetic and anti-adipogenic effects (normalisation of serum glucose, cholesterol, triacylglycerols, non-esterified fatty acids, insulin, adiponectin, and soluble intercellular adhesion molecule-1) [99].

### 5.6. Impact of Green Tea on Gut Microbiota

The polyphenols in green tea exist in complex oligomeric structures and glycosides. They exhibit low bioavailability in the small intestine [71], and their absorption involves the gut microbiota, primarily *Flavonifractor plautii*, *Slackia equolifaciens*, *Slackia isoflavoniconvertens*, *Adlercreutzia equolifaciens*, *Eubacterium ramulus*, *Eggerthella lenta*, *Lactobacillus* spp., and *Bifidobacterium* spp. [118]. Polyphenol metabolism occurs in three stages: (1) hydrolysis by intestinal enzymes and gut microflora; (2) metabolism in the liver; and (3) absorption of metabolites produced by sulfonate, glucuronide, and catechol transferase activities. Polyphenols influence the growth and metabolism of gut bacteria, thereby demonstrating their prebiotic effects. This prevents damage to the gut barrier and balances the synthesis of pro-inflammatory and anti-inflammatory T lymphocytes [118,119]. In vitro studies using human Caco-2 cells have shown that polyphenols from green tea stimulate the growth of *Bifidobacterium*, *Lactobacillus*, and *Enterococcus* spp. [112]. Glycans resulting from the breakdown of glycosidic bonds in polyphenols are utilised by bacteria that produce β-glucuronidase, such as *Bacteroides* and *Bifidobacterium* [71,120]. Specific phenolic compounds also influence the composition of the microbiota. Catechins from tea have been shown to inhibit the growth of *Bacillus cereus*, *Campylobacter jejuni*, *Clostridium perfringens*, *Escherichia coli*, *Helicobacter pylori*, *Legionella pneumophila*, and *Mycobacterium* spp. by damaging their cell membranes, particularly binding to peptidoglycans of Gram-positive bacteria, causing their disruption, which does not apply to Gram-negative bacteria [121]. Flavonols positively affect the ability of probiotic *Lactobacillus* to adhere to the intestinal mucosa [122]. Kaempferol improves intestinal barrier integrity and suppresses intestinal inflammation by reducing activation of the TLR4/NF-κB pathway and counteracts obesity-related dysbiosis [123], which is a risk factor for DILI.

## 6. Controversies

Some authors have suggested that consuming excessive amounts of green tea may have toxic effects on the liver owing to EGCG. Safe consumption is considered to be 2–3 cups of green tea per day containing about 250 mg of catechins [76], although the EFSA Panel [124] did not confirm that high doses of catechins could adversely affect liver function.

Studies conducted on rats have shown that in the context of clinical acetaminophen (paracetamol) overdose (2 g/kg, orally), administration of green tea extract (8.5 mg/kg, orally) likely exacerbates APAP-induced hepatotoxicity through oxidative stress and caspase 3-dependent apoptosis [24]. In experimental rats after one month, increased liver enzyme levels, necrosis and degeneration of liver cells, congestion, haemorrhage, inflammation, and fibrosis were observed. Other studies in rats have indicated that EGCG may induce hepatotoxicity by exacerbating pre-existing mitochondrial abnormalities, as EGCG can only penetrate mitochondrial membranes when their permeability is increased [13]. Gastric administration of EGCG (500–750 mg/kg) to C57BL/6J mice once daily for three days resulted in liver inflammation, necrosis, and haemorrhage [125]. EGCG-induced hepatotoxicity is associated with increased oxidative stress and decreased levels of SOD and GPX. These mice also exhibited reduced copies of liver mitochondria and decreased mRNA levels of the respiratory chain complex I and III marker genes, sirtuin 3, forkhead box O3a, and peroxisomes. Furthermore, in mice receiving an immortal toxic dose (75 mg/kg) of EGCG, repeated EGCG treatment significantly reduced the levels of exogenous antioxidants in the liver; whereas, a non-toxic dose (45 mg/kg) had no effect on these indicators, and a lethal dose (200 mg/kg) dramatically decreased the main antioxidant defence of the body [10]. Studies in mice have shown that, under selenium deficiency (which exhibits strong antioxidant properties), EGCG activates the hepatic Nrf2 response, leading to increased levels of heme oxygenase 1, NAD(P)H oxidoreductase 1, and thioredoxin activity [126]. This suggests that EGCG is a potent inducer of the Nrf2 system only under selenium-deficient conditions, whereas in mice with optimal selenium levels, thioredoxin and GSH systems serve as the first line of defence against high doses of EGCG-induced stress, sparing Nrf2 system activation [126].

However, results from another study conducted on mice suggested that EGCG may modulate its own bioavailability and that a diet containing EGCG could reduce the toxic impact of high oral bolus doses of EGCG (reducing EGCG levels in the liver by 71% and in serum by 57%) [127]. This finding may partially explain the variability observed in the hepatotoxic responses to dietary supplements containing green tea across different studies. Depending on the dose and biological system involved, EGCG may act either as an antioxidant or an inducer of antioxidant defence through its pro-oxidative action or other unidentified mechanisms.

## 7. Summary and Perspectives

Improving the effectiveness of DILI detection is crucial for enhancing patient safety and developing methods for assessing potential hepatotoxicity during drug development phases. Understanding the mechanisms underlying DILI will enable the development of strategies to mitigate its effects and minimise risks. Complete elimination of drugs is often not feasible, but supporting the body and liver through appropriate nutrition can help attenuate the adverse effects associated with DILI. Dietary therapy can serve as a simple yet effective approach to complement medical treatment. Polyphenols, particularly EGCG, found in green tea are promising because of their strong antioxidant, anti-inflammatory, and prebiotic properties.

The findings from studies conducted by various researchers are promising, albeit with some uncertainties. Therefore, future research should focus on elucidating the therapeutic and preventive mechanisms of polyphenols in liver health, including their interaction with the gut microbiota and epigenetic pathways, which significantly influence overall health. Studies conducted on obese mice have unequivocally shown the impact of bioactive food components (various parts of the watermelon fruit) on the expression of genes involved in processes such as xenobiotic metabolism in the liver [128]. Understanding the pathophysiological mechanisms of DILI is critical for the development of therapeutic and preventive strategies. Although many aspects of these mechanisms remain to be fully understood, it is widely recognised that factors such as direct toxic effects, oxidative stress, inflammation, mitochondrial damage, and immune reactions all contribute significantly to the development of DILI. Continued research in these areas will help refine our understanding and improve clinical outcomes for patients at risk of liver injury from medication.

One should also not forget about other components of green tea that have a proven protective effect on the liver, as their presence enhances the overall benefits of the tea. In particular, polysaccharides play a significant role in alleviating oxidative damage and inflammatory responses in liver tissues, preventing damage in non-alcoholic fatty liver disease and improving liver metabolism, as demonstrated in studies conducted on mice and rats [129,130].

The analysis of the available literature highlights certain limitations and gaps, particularly concerning clinical studies: (1) the presented studies involve relatively small patient groups, which may hinder the interpretation of the results; (2) the genetic factors of patients, which can influence the outcomes due to varying resistance and tolerance to medications, are often not considered; and (3) the structure of the patients’ gut microbiota, which plays a crucial role in overall body function and immunity, is frequently overlooked.

## Figures and Tables

**Table 1:** The impact of green tea on antioxidant parameters and liver function parameters—studies involving humans.

Characteristic	Duration of the Study	Disease	Dosage of Green Tea	Antioxidant Parameters	Liver Parameters	Type of Study	Reference
Control n = 40 Experimental n = 40	12 weeks	Non-alcoholic fatty liver disease	500 mg/day of GTE		↓ ALT, ↓ AST	Double-blind, placebo-controlled, randomized clinical trial	[51]
Control n = 40 Experimerntal n = 80	12 weeks	Non-alcoholic fatty liver disease	1000 mg/day of GTE		↓ ALT, ↓ AST, ↓ hs-CRP	Double-blind, placebo-controlled, randomized clinical trial	[52]
Control n = 20 Experimental n = 20	12 weeks	Moderate hypercholesterolemia	2 × 300 mL catechin-enriched green tea/day	↑ TEAC, ↑ GSH, ↑ SOD, ↑ CAT, ↑ GPx, ↑ GR	reverting mild fatty liver to the normal hepatic condition	Randomized, controlled trial	[53]
Control n = 12 Experimental n = 26	6 months	Non-alcoholic steatohepatitis	600 mg/day catechins		↓ ALT, ↓ AST	Double-blind, placebo-controlled, randomized clinical trial	[54]
Control n = 24 Experimental n = 21	3 months	Non-alcoholic fatty liver disease	550 mg/day green tea tablets		↓ AST	Placebo-controlled, randomized clinical trial	[55]
Control n = 16 Experimental n = 16	4 weeks	Operating room staff chronically exposed to inhalation anesthetics			↓ AST, ↓ ALT, ↓ ALP, ↓ bilirubin	Placebo-controlled, randomized clinical trial	[56]

↑—Increased concentration or activity in comparison to the control (healthy) group; ↓— decreased or inhibited concentration or activity in comparison to the control (healthy) group; ALT—alanine aminotransferase; AST—aspartate aminotransferase; ALP—alkaline phosphatase; CAT—catalase; GTE—green tea extract; GSH—glutathione; GPx—glutathione peroxidase; GR—glutathione reductase; hs-CRP—high-sensitivity *C*-reactive protein; SOD—superoxide dismutase; and TEAC—trolox equivalent antioxidant capacity.

**Table 2:** The impact of green tea on antioxidant parameters and liver function parameters—studies involving laboratory animals.

	Animal Species	Duration of Experiment	Treatments	Dosage of Green Tea	Antioxidant Parameters	Liver Parameters	Anti-inflammatory Indices	Tissues	References
Control n = 6 Experimental n = 18	Male Wistar rats	28 days	Intraperitoneal injections of *N*-nitrosodimethylamine in a dose of 1 mg/100 g body weight on 3 consecutive days of a week	0.2 mg EGCG/100 g body weight	↓ MDA	↓ ALT, ↓ AST		Serum	[57]
Control n = 5 Experimental n = 25	Male and female mice ICR	7 days	Stress-induced liver injury and immunosuppression	40 mg EGCG/kg		↓ ALT, ↓ AST	↓ IL-1β, ↓ IL-2, ↓ IL-6	Serum, liver	[58]
Control n = 6 Experimental n = 22	Female Sprague–Dawley rats	8 weeks	Non-alcoholic fatty liver disease	50 mg EGCG/kg	↓ iNOS, ↓ COX-2, ↓ TNF-α	↓ ALT,↓AST ratio, ↓ number of fatty score, necrosis	↓ inflammatory foci	Serum, liver	[59]
Control n = 8 Experimental n = 32	Male C57BL/6 mice	4 weeks	Methionine- and choline-deficient diet-induced non-alcoholic steatohepatitis	25, 50, or 100 mg EGCG/kg		↓ ALT, ↓ AST		Serum	[60]
Control n = 5 Experimental n = 30	Female C57BL/6 mice	4 days	CCl_4_-induced liver injury	100 mg GTE/kg		↓ ALT, ↓ AST, ↓ liver index		Serum, liver	[61]
Control n = 6 Experimental n = 18	Male ICR mice	7 days	Lipopolysaccharide-induced inflammatory liver injury	100 or 200 mg green tea polyphenols/kg body weight	↓ MDA, ↓ GSH, ↑ SOD	↓ ALT, ↓ AST	↓ IL-1β, ↓ IL-18, ↓ IL-6, ↓ TNF-α	Serum, liver	[62]
Control n = 6 Experimental n = 24	Male Sprague–Dawley rats	2 months	Thioacetamide-induced liver injury	250 mg/kg or 500 mg/kg daily methanolic GTE	↓ MDA, ↑ SOD, ↑ CAT	↓ ALT, ↓ AST, ↓ ALP, ↓ bilirubin		Serum, liver	[63]
Control n = 12 Experimental n = 36	Male C57BL/6J mice	4 weeks	Methionine–choline-deficient diet-induced non-alcoholic steatohepatitis	50 mg/kg EGCG		↓ ALT		Serum	[64]
Control n = 12 Experimental n = 6	Male C57BL/6J mice	14 weeks	High-fat diet-induced non-alcoholic fatty liver disease	EGCG—50 mg/kg/day	↓ ROS, ↑ GPx, ↑ SOD, ↑ CAT	↓ ALT, ↓ AST		Serum, liver	[65]
Control n = 12 Experimental n = 48	Adult male Wistar rats	6 or 12 weeks	7 mg CdCl2 + 50 mg Pb(CH_3_COO)_2_ per kg of feed	Green tea infusion (contains 111 mg tannic acid) per 1000 mL H_2_O_2_	↑ SOD, ↑ CAT, ↑ GPx			Liver	[11]
Control n = 20 Experimental n = 23	Male Nrf2-null mice, male C57BL6 WT mice	8 weeks	High-fat diet-induced non-alcoholic steatohepatitis	2% GTE	↓ MDA	↓ ALT	↓ TNF-α	Liver	[66]
Control n = 8 Experimental n = 24	Male Wistar rats	1 week	Halathion 150 mg/kg by gavage	30 mg/kg green tea through intraperitoneal injection	↓ LPO, ↑ TAP, ↑ TTG	↓ ALT, ↓ AST, ↑ ChE		Plasma, liver	[67]
Control n = 20 Experimental n = 40	Adult mice Balb-C strain	12 weeks	High-fat and high-cholesterol diet-induced hepatic steatosis	1% green tea over in food		↓ ALT, ↓ AST, ↓ ALP		Serum	[68]
Control n = 10 Experimental n = 30	Male Kunming mice	12 weeks	D-galactose-induced liver ageing	0.05% green tea polyphenols diet	↑ SOD, ↑ CAT, ↑ GSH, ↑ GST, ↑ TAC, ↓ MDA, ↓ NO	↓ ALT, ↓ AST, ↓ ALP	↓ TNF-α, ↓ TGF-β, ↓ IL-1β, ↓ IL-6		[69]

↑—Increased concentration or activity in comparison to the control (healthy) group; ↓—decreased or inhibited concentration or activity in comparison to the control (healthy) group; ALT—alanine aminotransferase; AST—aspartate aminotransferase; ALP—alkaline phosphatase; CAT—catalase; COX-2—cyclooxygenase-2 inhibitor; GSH—glutathione; GPx—glutathione peroxidase; iNOS—inducible nitric oxide synthase; NO—nitric oxide; ROS—reactive oxygen species; SOD—superoxide dismutase; IL-1β—interleukin-1 beta; IL-2—interleukin 2; IL-6—interleukin 6; IL-18—interleukin 18; MDA—malondialdehyde; LPO—lipid peroxidation; TAP—total antioxidant power; TTG—total thiol group; TAC—total antioxidant capacity; GST—glutathione S-transferase; ChE—cholinesterase activity; Nrf2—nuclear factor erythroid 2-related factor 2; TNF-α—tumour necrosis factor alpha; TGF-β—transforming growth factor-beta; GTE—green tea extract; and EGCG—epigallocatechin gallate.
